# Carpal Tunnel Syndrome: A Comprehensive Review

**DOI:** 10.7759/cureus.93459

**Published:** 2025-09-29

**Authors:** Cezary Milczarek, Bartosz Czyzewski, Karol Kozlowski, Wojciech Zywiec, Alicja Dorota, Michal Dorota, Illia Koval, Joanna Czyzewska, Oliwia Kawa

**Affiliations:** 1 Medicine, Medical University of Lodz, Łódź, POL; 2 Medicine, Central Clinical Hospital of the Medical University of Lodz, Łódź, POL; 3 Anesthesia and Critical Care, Provincial Integrated Hospital in Kielce, Kielce, POL; 4 Medicine, Provincial Hospital of St. Luke in Tarnów, Tarnów, POL; 5 Medicine, Zaglebiowskie Oncology Center in Dąbrowa Górnicza, Dąbrowa Górnicza, POL; 6 Conservative Dentistry, Central Clinical Hospital of Medical University of the Lodz, Łódź, POL; 7 Medicine, Provincial Hospital in Poznań, Poznań, POL; 8 Rheumatology Clinic, Medical University of Lodz, Łódź, POL

**Keywords:** carpal tunnel release, carpal tunnel syndrome, entrapment neuropathy, median nerve, neuropathy

## Abstract

Carpal tunnel syndrome (CTS) is the most common peripheral entrapment neuropathy, arising from chronic compression of the median nerve within the carpal tunnel. It significantly reduces quality of life and work performance, particularly among individuals exposed to repetitive wrist movements, and is more prevalent in women between the ages of 40 and 60. Typical clinical manifestations include nocturnal paresthesias, progressive sensory deficits, and weakness of the thenar muscles, all of which impair daily hand function. Diagnosis relies on clinical evaluation, provocative tests, electrophysiological studies, and increasingly on imaging techniques, such as ultrasound, which offer high sensitivity and specificity. A variety of treatment strategies are available, ranging from conservative measures, including splinting, corticosteroid therapy, manual therapy, acupuncture, and extracorporeal shock wave therapy, to surgical interventions aimed at decompression of the median nerve. While non-surgical methods provide meaningful relief in early or mild cases, surgery remains the gold standard in advanced or refractory disease. Minimally invasive approaches, including endoscopic and ultrasound-guided release, as well as the WALANT (Wide Awake Local Anesthesia No Tourniquet) technique for anesthesia, represent recent advances that improve recovery time, reduce complications, and enhance patient satisfaction. CTS continues to present a significant clinical and socioeconomic challenge, and optimal management requires timely diagnosis and a tailored, evidence-based therapeutic approach.

## Introduction and background

Carpal tunnel syndrome (CTS) is the most common entrapment neuropathy. This condition is well-recognized among clinicians. CTS represents the most prevalent type of entrapment neuropathy, which refers to peripheral nerve injuries caused by chronic compression within anatomically constrained spaces [1]. Due to the high prevalence of CTS and its impact on quality of life and the economy, it is essential to ensure appropriate management of the condition. This review aims to provide a clear and structured overview of the core aspects of CTS, as well as to present and analyze the available diagnostic and therapeutic strategies, with particular emphasis on recent advancements and innovative approaches that have improved both diagnostic accuracy and treatment outcomes.

## Review

Epidemiology and risk factors

The prevalence varies depending on the diagnostic criteria used, ranging from 14.4% among individuals reporting typical symptoms to 4.9% in cases confirmed by electrophysiological testing [2]. Higher prevalence rates have been observed in occupational groups exposed to repetitive hand movements and mechanical stress, particularly those operating vibrating tools, performing assembly line tasks, working in food processing, and engaging in packaging activities [3]. CTS is also more prevalent in females, especially between the ages of 40 and 60, with symptom onset occurring significantly earlier in females compared to males [4]. Numerous medical conditions contribute to an increased prevalence of CTS. These include pregnancy, hypothyroidism, diabetes mellitus, and certain rare disorders, such as mucopolysaccharidoses, mucolipidoses, and acromegaly, as well as a history of distal radius fractures [5-10].

Clinical features

The initial symptoms of CTS typically include nocturnal paresthesias. Over time, however, the paresthesias become more persistent, gradually increasing in both intensity and frequency, and eventually start to occur during the daytime as well. As the condition advances, patients begin to experience more pronounced sensory deficits in the median nerve distribution, including a diminished ability to feel light touch or temperature. In parallel, motor symptoms emerge, notably weakness of the thenar muscles, which impairs the coordination and strength of the thumb, further limiting hand function in everyday tasks [11]. All symptoms of CTS manifest within the sensory and motor territory of the median nerve. This nerve provides innervation to the palmar surface of the thumb, index finger, middle finger, and the radial (lateral) half of the ring finger. In addition to its sensory function, the median nerve also innervates the thenar muscles, which are crucial for performing fine motor movements involving the thumb, specifically opposition, abduction, and flexion. These muscle functions are essential for a wide range of everyday manual tasks. Consequently, when the nerve is compressed and its function compromised, patients commonly experience a combination of numbness, tingling, and muscle weakness in the affected hand. These impairments significantly disrupt day-to-day activities. Many patients report increasing difficulty with basic manual tasks such as gripping and holding objects securely, fastening buttons on clothing, or opening jars and bottles. Over time, these limitations can reduce independence, affect occupational performance, and diminish overall quality of life [12].

Diagnosis

There are numerous clinical signs and maneuvers utilized in the diagnostic assessment of CTS, each demonstrating variable levels of diagnostic accuracy in terms of sensitivity and specificity. Among the most frequently employed provocative tests are the wrist flexion test, commonly known as Phalen’s test, and the percussion test, referred to as the Hoffmann-Tinel sign. These diagnostic tools are designed to provoke or amplify the characteristic symptoms associated with median nerve compression, thereby aiding in clinical confirmation of the condition. Phalen’s test involves instructing the patient to maintain maximal wrist flexion for a period of time, typically up to 60 seconds, with the goal of reproducing the paresthesias or discomfort within the median nerve distribution. This test has been reported to have a relatively high sensitivity of approximately 85% and a specificity of 89%, making it a reliable component of the physical examination. Conversely, the Hoffmann-Tinel sign involves gentle percussion over the region of the transverse carpal ligament. A positive test is indicated by the elicitation of tingling or electric shock-like sensations radiating into the fingers innervated by the median nerve. This sign, while clinically useful, is associated with a somewhat lower sensitivity of 67% and specificity of 68% [13].

Another diagnostic maneuver that deserves mention due to its impressive specificity is the hand elevation test. In this procedure, the patient is asked to raise both hands above the level of the head and to maintain that position for approximately one minute. The reappearance or exacerbation of symptoms during this posture is interpreted as a positive result. The test has demonstrated a noteworthy sensitivity of 75.5% and an exceptional specificity of 98.5%, suggesting its utility as a highly confirmatory measure when evaluating suspected cases of carpal tunnel syndrome [14].

It is also worth emphasizing the diagnostic tools in the form of scales and questionnaires that are frequently used in both clinical practice and research. Among the most widely recognized and commonly applied are the CTS-6 clinical diagnostic tool, the Kamath and Stothard score, and the Boston Carpal Tunnel Questionnaire. These instruments are often utilized to support clinical decision-making and provide a structured assessment of the symptoms, functional limitations, and overall severity of the condition. Despite their popularity and frequent inclusion in diagnostic protocols, it is important to note that current evidence does not offer strong or definitive support for their effectiveness in significantly improving diagnostic accuracy. As such, while they may offer some supplementary value in assessing patient-reported symptoms and functional impact, their role in confirming the diagnosis of carpal tunnel syndrome remains limited and should be interpreted with caution in isolation [15]. In addition, several sensory and motor function tests exist, but according to the literature, none of them demonstrate sufficient sensitivity or specificity to be reliably used by clinicians for confirming the diagnosis. Among the available options, only those tests that demonstrate very high specificity, such as hand grip strength, pinch grip strength, and the presence of thenar atrophy, are regarded as clinically meaningful indicators [16]. One of the most important tests, especially when surgical intervention is being considered, is electrophysiological examination such as electroneurography (ENG) or electromyography (EMG). ENG, which assesses the prolonged latency of the median nerve affected by the syndrome, has been shown to have a sensitivity of over 85% and a specificity exceeding 95%. However, to minimize the risk of false-positive results, it is recommended to perform at least two separate measurements. Careful consideration should be given to individual patient-related factors that may influence the results, such as age-related physiological changes and variations in body mass index, both of which can affect nerve conduction parameters [17].

In recent years, significant advances have been made in imaging techniques. Ultrasound (US) has become a widely accessible diagnostic tool, and improvements in equipment specifications now allow for better image visualization. As a result, ultrasound has increasingly been used in the diagnosis of CTS. One of the most useful parameters is the cross-sectional area (CSA) of the median nerve; a CSA of the median nerve greater than 10 mm² allows for the diagnosis of CTS with a sensitivity of 82% and a specificity of 87%. However, to increase diagnostic certainty, it should be combined with other parameters. Other sonographic parameters include the assessment of nerve echogenicity, the presence of the notching or reversed notch sign, and increased vascularity observed in color Doppler imaging. According to the literature, increased vascularization tends to occur in the early stages of the syndrome but diminishes as the condition becomes chronic [18,19]. In a separate blinded study that carefully compared the effectiveness of two commonly used diagnostic methods, US and EMG, researchers found no significant difference in their ability to accurately diagnose CTS. Both techniques demonstrated comparable diagnostic performance, suggesting that either could be reliably used in clinical settings. However, despite this equivalence in effectiveness, US emerged as the preferred choice among clinicians and patients alike. This preference can be attributed to several practical advantages offered by ultrasound, including its generally lower cost, which makes it more accessible to a wider range of healthcare facilities and patients. Additionally, US is non-invasive and painless, which contributes to a more comfortable experience for patients during the examination. These factors combined make ultrasound a particularly attractive option in the diagnostic evaluation of carpal tunnel syndrome [20].

Additional diagnostic methods include shear wave elastography and high-definition (HD) color imaging, which utilizes B-flow visualization. Studies investigating the clinical utility of elastography have revealed a correlation between increased stiffness of the median nerve and the severity of CTS, potentially aiding in treatment decision-making. These findings were compared with results from electrophysiological studies, and a stiffness cutoff value of 50.12 kPa was proposed. Furthermore, HD color imaging has demonstrated greater sensitivity than conventional Doppler ultrasound. However, patients with diabetes were excluded from this study due to multifactorial causes of CTS, and thus the reported results do not apply to this group [21].

Another imaging modality useful in the diagnosis of CTS is magnetic resonance imaging (MRI). It provides information on morphological changes of the median nerve such as swelling, altered signal intensity, or bowing of the flexor retinaculum. Compared to ultrasound, MRI is also less operator-dependent. However, it should be noted that MRI is not yet a routine diagnostic tool and is typically reserved for more complex cases, as none of the described parameters alone can reliably confirm the presence of carpal tunnel syndrome [22].

Treatment

The treatment of CTS can be divided into non-surgical and surgical approaches. In the following section, we aim to present the available methods and discuss their effectiveness.

Non-surgical management can be further categorized into pharmacological and non-pharmacological approaches.

Pharmacological treatment includes the use of oral analgesics and anti-inflammatory medications, glucocorticosteroids, as well as corticosteroid injections administered into the carpal tunnel region. A review analyzing 16 studies on pharmacological treatment showed that corticosteroid use, including even a short two-week course of oral administration, was effective in providing short-term symptom relief. Locally injected corticosteroids provided more rapid symptom improvement; however, over longer follow-up periods, no significant advantage over oral administration was observed [23].

Among the various manual therapy options for CTS, nerve gliding exercises are particularly common and frequently recommended. These exercises aim to facilitate smooth movement of the median nerve within the carpal tunnel, potentially reducing irritation and improving symptoms. In one study, patients were asked to perform these specific nerve mobilization movements over a six-week period, while also wearing a night splint. The purpose was to evaluate whether combining exercises with splinting would lead to better outcomes. The results demonstrated that there was no significant difference in symptom improvement between the group that did nerve gliding exercises alongside splint use and the group that only used the night splint. However, both groups experienced a noticeable reduction in symptom severity, indicating that the night splint alone contributed substantially to symptom relief, while the added benefit of nerve gliding exercises was not statistically proven. It should be noted that it is only a single randomized trial, and more studies, ideally a meta-analysis, are needed to determine its true clinical utility [24].

One of the non-surgical, conservative approaches used in the management of CTS is acupuncture. This technique, rooted in traditional Chinese medicine and developed over 3,000 years ago, involves the precise insertion of very fine needles into specific anatomical points on the body [25]. A meta-analysis investigating the efficacy of acupuncture in treating CTS, both as an independent intervention and in conjunction with other therapeutic modalities such as night splints or pharmacological treatment, indicated that acupuncture, when applied as an adjunctive therapy, may contribute to alleviating symptom severity, with particular benefit observed in pain reduction. Reported adverse effects are infrequent and generally mild in nature, most commonly manifesting as localized bruising at the needle insertion sites. In rare cases, patients may experience transient sensations such as tingling, numbness, or a short-term intensification of pain symptoms. However, it should be noted that overall evidence quality is limited, and further high-quality studies are needed to confirm its clinical effectiveness [26].

Much attention in recent years has been directed toward the potential application of extracorporeal shock wave therapy (ESWT) as a supportive modality in the treatment of carpal tunnel syndrome. This method, which involves the transmission of focused acoustic pressure waves to affected tissues, aims to induce a series of physiological responses, including the stimulation of tissue repair processes, attenuation of local inflammatory reactions, and enhancement of microcirculatory dynamics. Clinical data derived from a randomized controlled study indicate that ESWT may contribute to a meaningful reduction in the intensity of pain experienced by patients undergoing this intervention. Furthermore, a positive impact has been observed with respect to functional parameters, particularly improvements in hand grip strength, as well as measurable changes in the cross-sectional area of the median nerve, findings that suggest a favorable response to this therapeutic approach within selected patient populations [27].

Currently utilized surgical techniques for the treatment of CTS are primarily focused on achieving effective decompression of the median nerve by transecting the transverse carpal ligament, which is the primary anatomical structure responsible for compressive symptoms. This surgical objective can be accomplished through a variety of procedural approaches, each differing in invasiveness, incision size, and recovery time.

The most traditional and widely established method is the open surgical technique, which involves a larger incision to allow direct visualization of the operative field. In contrast, the endoscopic carpal tunnel release method utilizes smaller incisions and specialized instruments to access and divide the ligament with the assistance of an endoscopic camera, thereby minimizing tissue trauma. A third option is the limited open incision technique, which aims to strike a balance between direct visualization and reduced surgical exposure, offering a compromise between the open and endoscopic approaches.

An important clinical question that continues to be the subject of ongoing discussion is which surgical technique should be considered superior in the treatment of carpal tunnel--the endoscopic approach (employing either one or two portals) or the traditional open method. Both surgical modalities have been demonstrated to provide significant and lasting relief from pain and other symptoms associated with the condition. Nevertheless, the endoscopic technique tends to result in a more rapid alleviation of symptoms, especially regarding pain intensity and muscular weakness. Additionally, it is often linked to an earlier return to occupational and daily activities, although this advantage is balanced by the typically longer duration required to perform the procedure [28]. Short-term complications are observed more frequently following endoscopic surgery, with the most typical among them being transient neurapraxia. This condition, although usually self-limiting and reversible, represents a common concern in the early postoperative period. On the other hand, in the case of classical open procedures, there is a noticeably higher incidence of complications specifically related to the surgical wound. These wound-related issues are more frequently encountered in open surgery compared to endoscopic techniques and are considered an important factor when evaluating the overall morbidity associated with the traditional approach [11].

The newest and highly promising approach to CTS is ultrasound-guided carpal tunnel release (UCTR). This technique involves a skin incision of only 1 to 3 mm, as compared to 4-5 cm in the classic open method, 2 cm in mini-open surgery, and 1.5-2 cm in endoscopic techniques, and the introduction of surgical instruments under continuous ultrasound guidance. UCTR is associated with several notable advantages. It offers a faster return to hand function as compared to previously established techniques, primarily due to its less invasive nature and minimal tissue disruption. Additionally, the resulting postoperative scar is markedly smaller, which promotes more rapid healing and improved cosmetic outcomes. The use of ultrasound also reduces the risk of iatrogenic injury to nerves or blood vessels, as the surgeon can visualize and avoid these critical structures during the entire operation. Furthermore, the overall duration of the procedure is particularly brief; in one study, the average operative time was reported to be just 5.8 minutes, underscoring the efficiency of this novel method [29].

When considering the type of anesthesia administered during the surgical procedure, local anesthesia is typically the method of choice. Among the various techniques available, the WALANT (Wide Awake Local Anesthesia No Tourniquet) approach has become increasingly favored in clinical practice. This preference stems primarily from the numerous practical advantages it offers. Notably, patients undergoing WALANT anesthesia report significantly fewer postoperative symptoms, such as nausea and vomiting, which are commonly associated with general anesthesia. Moreover, this technique is associated with reduced overall healthcare costs, making it a more economical option for both healthcare providers and patients. In addition to these clinical and financial benefits, the WALANT method has also been linked to a higher degree of patient satisfaction, which further supports its growing adoption in surgical interventions, particularly in hand surgery [30].

One of the primary goals of surgical intervention in CTS is to alleviate symptoms. Interestingly, nerve conduction studies (ENG) performed after carpal tunnel release often show normalization of conduction parameters. However, the timeline for this recovery is not strictly defined; it may occur immediately following the procedure or extend over a period of up to 42 weeks. Notably, in some cases, despite technically successful surgery and complete resolution of clinical symptoms, ENG parameters may not return to normative values [31].

## Conclusions

CTS remains the most prevalent peripheral entrapment neuropathy, with a considerable burden on quality of life, occupational productivity, and healthcare resources. Optimal management requires a tiered, evidence-based approach, beginning with early recognition of risk factors and accurate diagnosis using both clinical tests and adjunctive modalities such as ultrasound, MRI, or electrophysiology. Conservative therapies, including wrist splinting, pharmacological agents, and selected adjuncts, such as acupuncture or extracorporeal shock wave therapy, provide meaningful benefit in early and mild disease, although their efficacy is generally short- to medium-term. Surgery remains the gold standard for moderate-to-severe or refractory CTS, with accumulating evidence supporting minimally invasive and image-guided approaches (e.g., endoscopic release, ultrasound-guided release, WALANT anesthesia) as safe and effective alternatives to conventional open release. Future research should prioritize long-term comparative outcomes, cost-effectiveness, and standardized patient-reported outcome measures to refine clinical decision-making. A structured diagnostic and treatment pathway, as outlined below, may guide clinicians toward timely and personalized management strategies.
